# Teneurin paralogues are able to localise synaptic sites driven by the intracellular domain and have the potential to form *cis*-heterodimers

**DOI:** 10.3389/fnins.2022.915149

**Published:** 2022-11-03

**Authors:** Angela Cheung, Greta Schachermayer, Aude Biehler, Amber Wallis, Mégane Missaire, Robert Hindges

**Affiliations:** ^1^Centre for Developmental Neurobiology, King’s College London, London, United Kingdom; ^2^MRC Centre for Neurodevelopmental Disorders, King’s College London, London, United Kingdom

**Keywords:** teneurin, synapse, protein interaction, cell adhesion molecule, dendritic spine

## Abstract

Synaptic specificity during neurodevelopment is driven by combinatorial interactions between select cell adhesion molecules expressed at the synaptic membrane. These protein–protein interactions are important for instructing the correct connectivity and functionality of the nervous system. Teneurins are one family of synaptic adhesion molecules, highly conserved and widely expressed across interconnected areas during development. These type-II transmembrane glycoproteins are involved in regulating key neurodevelopmental processes during the establishment of neural connectivity. While four teneurin paralogues are found in vertebrates, their subcellular distribution within neurons and interaction between these different paralogues remains largely unexplored. Here we show, through fluorescently tagging teneurin paralogues, that true to their function as synaptic adhesion molecules, all four paralogues are found in a punctate manner and partially localised to synapses when overexpressed in neurons *in vitro*. Interestingly, each paralogue is differentially distributed across different pre- and post-synaptic sites. In organotypic cultures, Tenm3 is similarly localised to dendritic spines in CA1 neurons, particularly to spine attachment points. Furthermore, we show that the intracellular domain of teneurin plays an important role for synaptic localisation. Finally, while previous studies have shown that the extracellular domain of teneurins allows for active dimer formation and transsynaptic interactions, we find that all paralogues are able to form the full complement of homodimers and *cis*-heterodimers. This suggests that the combinatorial power to generate distinct molecular teneurin complexes underlying synaptic specificity is even higher than previously thought. The emerging link between teneurin with cancers and neurological disorders only serves to emphasise the importance of further elucidating the molecular mechanisms of teneurin function and their relation to human health and disease.

## Introduction

The formation of precise synaptic connections between neurons during development is a fundamental process which ultimately dictates the correct functionality of the nervous system. Much research has been done on trying to unravel these complex mechanisms and pathways, focusing on different aspects including molecular, structural or activity-related processes. Protein interactions at the synaptic membrane play a pivotal role in driving synaptic specificity, for example, through cell adhesion molecules interacting in a combinatorial manner to generate diverse cellular interactions. While many cell adhesion molecules are implicated in this process, the teneurin family of type II transmembrane glycoproteins have been shown to be key mediators of intercellular signalling during development in both vertebrates and invertebrates.

Also known as Tenm/Odz, the teneurins were originally discovered in the early 1990s in *Drosophila* as tenascin-like molecule accessory (tena) and tenascin-like molecule major (tenm) (Baumgartner and Chiquet-Ehrismann, 1993; Baumgartner et al., 1994; Levine et al., 1994). Subsequent studies showed they are highly expressed across both the developing and adult nervous systems, particularly in interconnected regions (Oohashi et al., 1999; Cheung et al., 2019), reflecting a significant role in mediating basic neurodevelopmental processes, such as cell migration, axonal guidance, and synaptic partner matching (Drabikowski et al., 2005; Leamey et al., 2008; Hong et al., 2012; Berns et al., 2018; Del Toro et al., 2020; Pederick et al., 2021). While only one teneurin gene has been identified in most invertebrates, insects have two paralogues (Tucker et al., 2012) and four teneurin paralogues are present in vertebrates (Mosca, 2015). There is a high degree of conservation between paralogues, with 58–70% sequence identity between human teneurin-1 to -4 alone (Jackson et al., 2018).

Structurally, the teneurins are large proteins of around 300 kDa with a small N-terminal intracellular domain, a single span transmembrane domain, and a large C-terminal extracellular region (Rubin et al., 1999; Tucker and Chiquet-Ehrismann, 2006; Jackson et al., 2018; Li et al., 2018). While the small ∼45 kDa intracellular domain is less conserved than the extracellular region (Minet et al., 1999; Nunes et al., 2005), it nevertheless shows up to 70% sequence similarity between orthologues (Scholer et al., 2015). Shared features of the intracellular domain include an EF-hand-like Ca^2+^ binding site and polyproline-rich regions that can interact with SH3 domain-containing proteins, hypothesised to mediate interactions with the cytoskeleton (Nunes et al., 2005; Tucker and Chiquet-Ehrismann, 2006). Similarly, the C-terminal extracellular region is also composed of a number of domains, including epidermal growth factor (EGF)-like repeats, a cysteine-rich domain, a TTR (transthyretin-related) domain, a FN (fibronectin)-plug domain, five NHL (NCL-1, HT2A, and Lin-41) repeats, a YD (tyrosine-aspartate)-repeat domain, an internal linker domain, an ABD (antibiotic-binding domain-like) domain, the Tox-GHH domain, and a teneurin C-terminal associated peptide region (Qian et al., 2004; Wang et al., 2005; Jackson et al., 2018; Li et al., 2018; Cheung et al., 2019). The EGF-like repeats allow for the dimerisation of teneurin monomers in *cis* to form active dimers (Feng et al., 2002; Beckmann et al., 2013), while the NHL domains allow for transsynaptic interactions, either with teneurin itself, or with other cell adhesion molecules such as latrophilins (Boucard et al., 2014). The teneurin C-terminal associated peptide region, or TCAP, may be cleaved and released as a bioactive peptide and has even been shown to be able to interact with other protein receptors such as the latrophilins (Qian et al., 2004; Wang et al., 2005; Al Chawaf et al., 2007; Lovejoy et al., 2009; Woelfle et al., 2015).

In *cis*, active teneurin dimers can form as a result of both homophilic and heterophilic interactions between teneurin monomers. Electron microscope analysis showed that homophilic interactions are able to occur between the extracellular domains of all teneurins to form homodimers, while co-transfection experiments of cells with two different extracellular domains suggest teneurin heterodimers can also form (Feng et al., 2002). In *trans*, interactions can occur between teneurin proteins homophilically, or heterophilically with other protein families. Separate *in vitro* studies have shown that cell transfection expression constructs for with either *tenm2* or *tenm3* promotes homophilic cell-cell adhesion (Rubin et al., 2002; Beckmann et al., 2013; Berns et al., 2018), while *in vivo*, work in *Drosophila* show that the two teneurin paralogues, Ten-a and Ten-m, both contribute to synaptic specificity by interacting homo- and heterophilically in *trans* between select pairs of pre- and postsynaptic partners in the olfactory bulb and neuromuscular junction (Hong et al., 2012; Mosca et al., 2012; Mosca, 2015). Surprisingly, the *trans*-homophilic interactions between Tenm3 in promoting cell aggregation was dependent on the alternatively spliced isoform expressed (Berns et al., 2018). More recently, *trans*-synaptic heterophilic interactions between mammalian teneurins and latrophilins have been shown to be important in shaping synapse formation and neural connectivity (Boucard et al., 2014; Vysokov et al., 2016; Sando et al., 2019; Pederick et al., 2021).

Indeed, the disruption of teneurin function using *in vivo* models highlight the importance of teneurins during synaptic partner matching and the establishment of functional connectivity. For instance, the knockdown of *tenm3* in zebrafish leads to defects in retinal ganglion cell (RGC) and amacrine cell connectivity in the retina affecting, as a consequence, the functional development of a specific visual feature, orientation selectivity (Antinucci et al., 2013). Furthermore, studies in mouse have shown that the loss of Tenm2 and Tenm3 significantly affects the mapping of ipsilateral retinal projections to the superior colliculus (Leamey et al., 2007; Young et al., 2013). In addition to these studies focusing on the visual system, teneurins, together with their heterophilic interaction partners latrophilins, have been shown to regulate topographic circuit assembly between the CA1 region of the mouse hippocampus and the subiculum (Berns et al., 2018; Pederick et al., 2021). As such, with such an integral role in neurodevelopment and the establishment of connectivity in the brain, it is not surprising that mutations in all four human genes have also been linked to neurodevelopmental disorders (Mosca, 2015).

Although teneurins have been shown to play a significant role in regulating different aspects of neurodevelopment, much about their synaptic localisation, mechanism of action and role in synapse formation remains to be explored. The synthesis of synaptic proteins in neurons can occur in the soma, with proteins subsequently transported to synaptic sites, or alternatively, the mRNA is transported along axons and dendrites and locally translated in the vicinity of synaptic structures (Steward and Levy, 1982; Sutton and Schuman, 2006; Doyle and Kiebler, 2011; Donlin-Asp et al., 2021). However, it is so far not clear if teneurins undergo local translation or are transported in protein form to their site of action. Furthermore, despite the available data on *trans*-interactions, little is known about the extent of forming heterodimers in *cis* through their EGF domains, and thus increasing the molecular repertoire to control synaptic specificity. Here, we show the differential subcellular localisation of teneurin paralogues and further examine structural factors affecting its distribution in neurons. Furthermore, we show that all teneurin paralogues are able to interact with each other in *cis* and that there is a full complement of interactions. Our results set an important basis for future studies to shine light on the molecular diversity of synaptic protein complexes underlying synapse formation, specificity and function.

## Materials and methods

### Cortical neuronal culture and transfection

Dissociated neurons were generated from cortices of embryonic day 18 Sprague-Dawley rats of either sex. Dissected cortices were treated with 5 mg/ml trypsin (Thermo Fisher Scientific, UK) for 5 min at 37°C and triturated with fire-polished Pasteur pipettes. Neurons were plated at 80,000 viable cells/ml directly onto poly-L-lysine (100 μg/ml; Sigma-Aldrich, UK) coated sterile borosilicate glass coverslips for confocal imaging (18-mm diameter; Marienfeld, Germany). Cultures were maintained in Neurobasal A^®^ medium supplemented with 2% B27 supplement, 2% foetal bovine serum, 1% glutamax (all Thermo Fisher Scientific, UK) and 1% penicillin/streptomycin (Sigma-Aldrich, UK), at 37°C in a humidified incubator with 5% CO_2_. After 3 days *in vitro* (DIV) the medium was exchanged for serum-free media. At 7 DIV neurons were transfected with expression plasmids expressing EGFP, membrane-EGFP, LRRTM2-myc and EGFP/tagRFP-tagged full length mouse teneurin paralogues (tenm1_EGFP/tagRFP, tenm2_EGFP/tagRFP, tenm3_EGFP/tagRFP, tenm4_EGFP/tagRFP, tenm3ΔECD_EGFP, and tenm3ΔICD_EGFP) under the control of a CAG promoter using Lipofectamine 2000 transfection reagent (2-h incubation; Thermo Fisher Scientific, UK). After transfection neurons were maintained in serum-free medium until fixation with 4% paraformaldehyde (15 min; Sigma-Aldrich, UK) at 17 DIV.

### Organotypic hippocampal cultures

Organotypic hippocampal slices were acquired from CD1 mice sacrificed at postnatal day 7. Only schedule 1 procedures performed by a competent individual were used in these studies, which are exempt under the Animals Scientific Procedures Act 1986. After decapitation, the brains were removed and placed in a petri dish with ice-cold dissecting solution containing 23 mM D-glucose (Sigma-Aldrich, UK) in Gey’s balanced salt solution (GBSS, Sigma-Aldrich, UK). The hippocampi were dissected and placed on the Teflon stage of a tissue cutter, and coronal slices of 400 μm were cut and separated from each other by addition of dissecting solution and gentle mixing in a falcon tube. Well defined and undamaged slices were selected under the microscope and transferred onto sterile, porous (0.4 μm) Millicell-CM membranes (Merck Millipore) in six well tissue culture plates at a density of 4 slices per membrane. The slices were incubated at 35.5°C, 5% CO2 in 1.2 ml slice culture medium containing 49% Minimum essential medium (Thermo Fisher Scientific, UK), 25% Earle’s balanced salt solution (EBSS, Gibco), 25% heat inactivated horse serum (Thermo Fisher Scientific, UK), 1% B27 supplement and 35.4 mM D-glucose. At 1 DIV and every two days thereafter a full media change was done.

### DNA bullets and biolistic transfection

To make DNA bullets for biolistic transfection with a gene gun, 0.015 g of 1.6 μm gold microcarriers (Bio-Rad) were mixed with 100 μl spermidine (0.05 M, Alfa Aesar) and sonicated for 3 s. A total of 35 μg of *tenm3* expression vector and 10 μg of membrane-bound GFP expression vector, or 25 μg of membrane-bound GFP expression vector only, were added to the gold solution and vortexed. Then 100 μl of 1 M calcium chloride was added drop-wise to each of the gold-DNA solutions whilst vortexing. The gold microcarriers were left to precipitate for 10 min at room temperature and then centrifuged for 15 s. The supernatant was removed and 1 ml 100% ethanol added to the gold microcarriers, vortexed and centrifuged four times. Finally, 3 ml of 100% ethanol was added to the gold microcarriers and the gold-DNA solutions were vortexed and sonicated again for 3 s. The gold-DNA solutions were injected into 70 cm of tubing, which was previously rinsed with 100% ethanol and dried with nitrogen gas at 3–4 LPM flow for 20 min in a tubing prep station (Bio-Rad). The gold microcarriers were left to settle in the tube for 4 min before the ethanol was removed carefully and the tubing rotated by 180° and left for 4 s. Nitrogen was then passed through the tubing at 5 psi, 3 LPM whilst spinning for 5 min, the tubing cut using a clean razor blade and the DNA bullets stored at 4°C in tubes with silica gel. Organotypic slices from postnatal day 7 mice were transfected at 1 DIV. A Helios gene gun system (Bio-Rad) was used to shoot organotypic hippocampal slices with a helium pressure of ∼140 PSI (∼9.5 Bar). After transfection the slices were placed back into the incubator and gene expression and cell survival checked after 24 and 48 h.

### Immunofluorescence

Fixed cultures were washed in PBS before permeabilisation with 0.1% Triton X-100 in PBS (PBST) for 5 min. Cells were then washed again in PBS before non-specific antibody-binding sites were blocked by incubation in 5% goat serum (Sigma-Aldrich, UK) in PBST for 60 min at RT before incubation with appropriate dilutions of primary antibodies in PBST, 5% goat serum overnight at 4°C. Primary antibodies were used at the following concentrations: rabbit anti-synapsin I (1:500; Merck Millipore), mouse anti-bassoon (1:500; Abcam), mouse anti-shank2 (1:500; Neuromab), rabbit anti-vGLUT1 (1:500; GeneTex), rabbit anti-vGAT (1:250; GeneTex), mouse anti-PSD-95 (1:250; Thermo Fisher Scientific, UK), mouse anti-gephyrin (1:500; Synaptic Systems), chicken anti-GFP (1:500, Abcam), mouse anti-myc (1:200, Abcam), and rabbit anti-tagRFP (1:500, Invitrogen). Cells were washed three times in PBS before incubation with secondary antibodies in PBST for 2 h at RT, counterstained with Hoechst (Thermo Fisher Scientific, UK) and washed four times in PBS. Secondary antibodies were used at the following concentrations: Alexa Fluor goat anti-chicken 488, Alexa Fluor goat anti-mouse 488, Alexa Fluor goat anti-rabbit 568 and Alexa Fluor goat anti-mouse 568 (all used at 1:500; Thermo Fisher Scientific, UK). Cells were mounted using Fluoromount-G mounting medium (Thermo Fisher Scientific, UK).

### Confocal microscopy

Cultures were imaged on a Nikon A1R inverted confocal microscope (Nikon instruments, Melville, NY, USA) using a 60× Plan Fluor oil immersion objective (numerical aperture of 1.4). Excitation wavelengths of 405, 488, 561, or 640 nm were used. Images were acquired using NIS-Elements imaging software and were processed in image processing package FIJI (Schindelin et al., 2012).

The imaging of dendritic spines in organotypic slices was carried out using a Nikon A1R inverted confocal microscope with a 40× water immersion objective (NA 1.15) and 408-, 488, and 561 nm excitation wavelengths. Images were taken at 1× zoom for whole neuron morphology and 3× zoom for spine morphology and synaptic puncta (1× zoom = 0.31 μm/pixel, 3× zoom = 0.10 μm/pixel) and as a z-stack with 0.3 μm steps. Images were acquired using the NIS-Elements software and image stacks were exported as raw 16-bit ND2 files.

### HEK293 and neuro-2a cell culture

The human embryonic kidney cell line HEK293, and immortalised mouse neuroblastoma cell line Neuro-2a (N2a) was maintained in Dulbecco’s Modified Eagle’s Medium (DMEM, Thermo Fisher Scientific, UK) supplemented with 10% foetal bovine serum (Thermo Fisher Scientific, UK) and 1% penicillin/streptomycin (Sigma-Aldrich, UK), at 37°C in a humidified incubator with 5% CO_2_. Expression plasmids expressing EGFP-only and EGFP- or tagRFP-tagged teneurin paralogues (full length teneurin constructs or tenm3_ΔEGF_EGFP) were transfected into HEK293 or N2a cells using Lipofectamine 2000 transfection reagent and Opti-MEM reduced serum medium (3-h incubation; Thermo Fisher Scientific, UK). After transfection, HEK293 or N2a cells were maintained in complete media before fixation with 4% paraformaldehyde (15 min; Sigma-Aldrich, UK) and immunostaining or co-immunoprecipitation studies.

### Puncta analysis and statistical analysis

Puncta colocalisation analysis was carried out on images in FIJI using the Puncta Analyzer plugin developed and described (Ippolito and Eroglu, 2010). Puncta sizes below 0.1 μm and above 10 μm were excluded from the analysis. Plots were based on the mean of at least 12 cells per condition pooled from a minimum of three independent experiments. Data were plotted using GraphPad Prism data analysis software (GraphPad Software Inc., San Diego, CA, USA) and the appropriate statistical test (one-way ANOVA with Dunnett’s multiple comparison *post hoc* test) applied to the means.

### Co-immunoprecipitation and Western blot analysis

Expression vectors containing EGFP-tagged teneurin paralogues were co-transfected with tagRFP/Myc-tagged teneurin paralogues into Neuro2A cells with Lipofectamine 2000, the cell lysates collected after 2 DIV and immunoprecipitated with GFP-Trap agarose beads (ChromoTek, Germany). An EGFP-only vector was used as the bait control and co-transfected with the prey tagRFP/Myc-tagged teneurin paralogues. The precipitated products were separated using SDS-PAGE gel electrophoresis, transferred onto PVDF membrane and blocked for 1 h with 5% milk powder in TBS-Tween 20 (TBST) blocking solution before incubating overnight with primary antibody in blocking solution at 4°C. After primary incubation, blots were washed three times in TBST before incubation with secondary antibody in blocking solution for 2 h at RT. Blots were washed another three times in TBST before developed using Novex ECL chemiluminescent substrate reagent kit (Thermo Fisher Scientific, UK) and visualised on the Odyssey Fc imaging system (LI-COR Biosciences, UK). Blots were chemically stripped and re-probed with different antibodies. Antibodies were used at the following concentrations: Primaries mouse anti-myc tag (1:1,000; Cell Signaling) and chicken anti-GFP (1:5,000; Abcam), and secondaries goat anti-mouse IgG HRP conjugate (1:5,000; Abcam) and goat anti-chicken IgY HRP conjugate (1:5,000; Abcam).

## Results

### Teneurins do not evenly distribute along the membrane but are partially localised to puncta at synaptic sites

**FIGURE 1:**
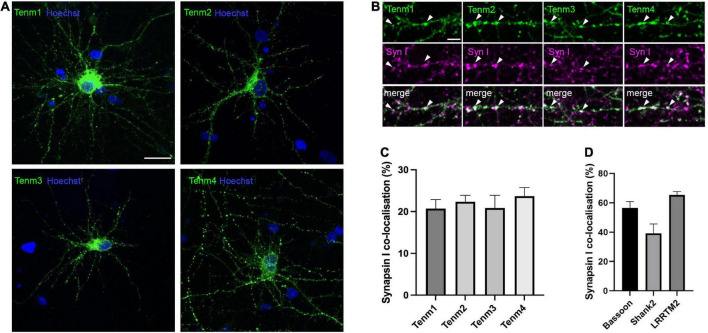
Teneurins form discrete puncta, some of which are localised to synaptic sites. **(A)** EGFP-tagged teneurin paralogues overexpressed in rat cortical cultures are unevenly distributed across the membrane. **(B)** Synapsin I immunostaining in dissociated cortical cells overexpressing EGFP-tagged teneurin show partial co-localisation of teneurin puncta with synapsin I (two examples marked with arrow heads for each teneurin member). **(C)** Similar levels of partial co-localisation are observed between all four teneurin paralogues with synapsin I. **(D)** Synapsin I co-localisation for bona fide synaptic proteins bassoon (56%), shank (38%), and LRRTM2 (65%). Data represents mean ± SE based on samples with at least 14 cells/condition pooled from a least three different experiments. One-way ANOVA with Dunnett’s multiple comparison test was performed between all paralogues (*p* < 0.05). Scale bar, 20 μm **(A)**, 5 μm **(B)**.

To investigate the subcellular localisation pattern of all four teneurin paralogues in neurons *in vitro*, we used dissociated primary neuronal cultures. Due to a lack of reliable antibodies for all teneurins, we transfected neurons with expression constructs for EGFP-tagged mouse teneurins at 7 DIV and analysed the cultures at 17 DIV, when synaptic connections are formed and matured. Teneurin insertion into the membrane in HEK293 cells was unaffected by the EGFP-tag (Supplementary Figure 1). In dissociated neurons, all four teneurins were found to form discrete puncta along both dendrites and axons, instead of being evenly distributed along the membranes as seen in HEK cells (Figure 1A). To assess a possible co-localisation of the detected teneurin puncta with the presence of synapses, we used specific antibodies against the general marker synapsin I. We found that all four teneurins partially co-localise with synapsin I (Figure 1B), with no significant difference in the proportion between Tenm1 (21%), Tenm2 (22%) Tenm3 (21%), and Tenm4 (24%) (Figure 1C, *n* = 14–16). The values are generally slightly lower than for our controls assessing synapsin I co-localisation with other bona fide synaptic proteins bassoon (56%), shank 2 (38%) or LRRTM2 (65%) (Figure 1D), however we find good co-localisation of LRRTM2 with teneurins as seen in one example for Tenm3 (Supplementary Figure 2). Rotation of one image channel by 90° and assessing its puncta co-localisation with the synapsin I puncta as a control showed values consistently below 4% (example shown in Supplementary Figure 3).

### Teneurins are differentially distributed across inhibitory and excitatory pre- and postsynaptic sites

**FIGURE 2:**
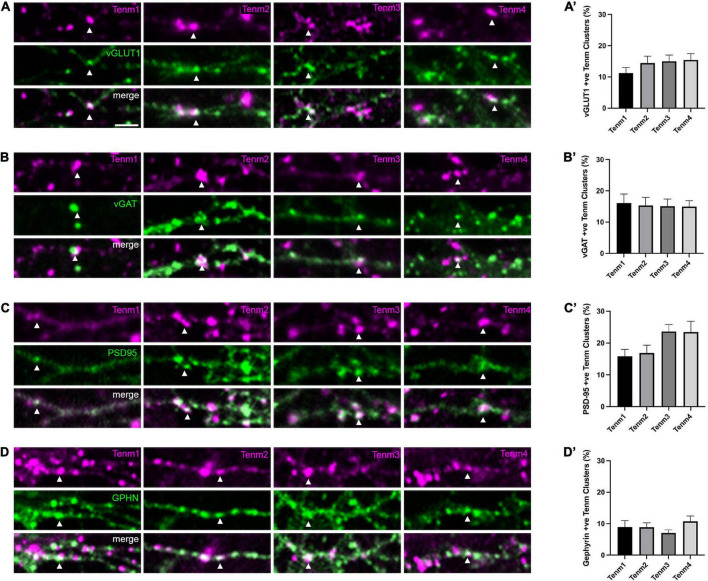
Teneurin distribution across different synaptic sites. Immunocytochemistry detection of **(A)** vGLUT, **(B)** vGAT, **(C)** PSD95, and **(D)** Gephyrin in dissociated neurons transfected with EGFP-tagged teneurin paralogues. In each panel, one example colocalisation is indicated by the arrowhead. Graphs on the right show quantification of colocalisation. **(A′–D′)** Levels of teneurin colocalisation with vGLUT, vGAT, and PSD-95 are similar (10–20%), while co-localisation with Gephyrin is slightly lower (below 10%). Data represents mean ± SE based on samples with at least 12 cells/condition pooled from a least three different experiments. One-way ANOVA with Dunnett’s multiple comparison test was performed between all paralogues (*p* < 0.05). Scale bar, 5 μm (all panels).

To further investigate the distribution of teneurin paralogues, we assessed teneurin co-localisation with specific types of synapses. To distinguish between excitatory and inhibitory synapses and their pre- and postsynaptic components, we used an immunocytochemistry approach using antibodies against the vesicular glutamate transporter vGLUT (Figure 2A) and the vesicular GABA transporter vGAT (Figure 2B), as well as postsynaptic density protein 95 (PSD-95, Figure 2C) and gephyrin (Figure 2D), respectively. Overall, we found that all four teneurin paralogues partially co-localise with all these markers in various proportions (Figures 2A’–D’).

For presynaptic compartments, our results show that roughly 13% of teneurin clusters were also positive for vGLUT [Tenm1 (11%), Tenm2 (14%), Tenm3 (15%), and Tenm4 (15%), *n* = 14–15] and there was no significant difference in the distribution of teneurin paralogues across excitatory presynaptic sites (Figure 2A’). Similarly, around 15% of teneurin clusters were also positive for vGAT [Tenm1 (16%), Tenm2 (15%), Tenm3 (15%), and Tenm4 (15%), *n* = 12–17], again with no significant difference observed between the presence of different teneurin paralogues at inhibitory presynaptic termini (Figure 2B’). Similar to the presynaptic compartments, we found all teneurins to be present in both excitatory and inhibitory postsynaptic compartments. Co-localisation with excitatory postsynaptic PSD-95 was detected in Tenm1 (16%), Tenm2 (17%), Tenm3 (24%), and Tenm4 (23%) (Figure 2C’, *n* = 16–17). Finally, the localisation of teneurin paralogues to inhibitory postsynaptic sites, as labelled by gephyrin, was the lowest out of all the synaptic sites with just over 9% of teneurin clusters also gephyrin positive (Tenm1 (9%), Tenm2 (9%), Tenm3 (7%), and Tenm4 (11%) and no significant difference observed between paralogues (Figure 2D’, *n* = 14–17). From these experiments we conclude that, despite the rather low percentages, teneurins are generally able to localise to pre- and postsynaptic compartments, both in excitatory and inhibitory cells.

### Teneurin 3 is localised to spine heads and spine attachment points in CA1 neurons

**FIGURE 3:**
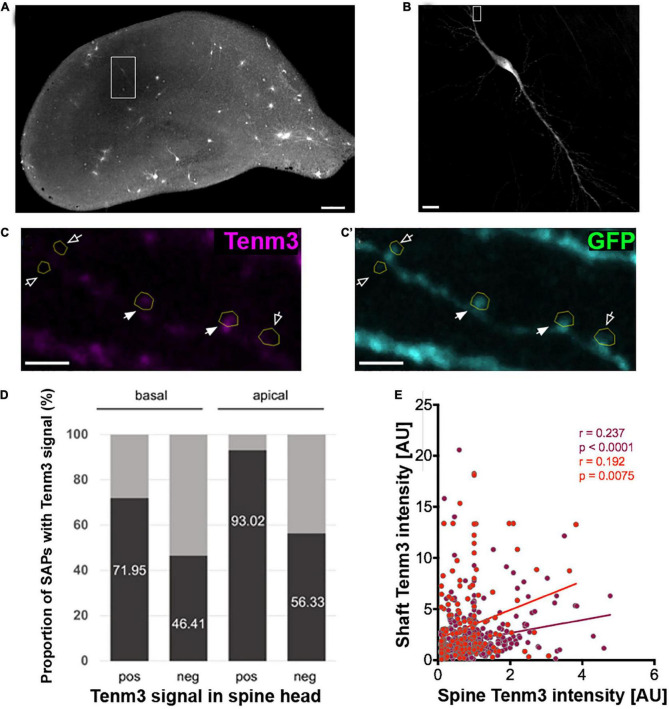
Tenm3 is localised to SAPs in CA1 neurons. **(A)** Organotypic hippocampal slice biolistically transfected with Tenm3 and GFP. White square indicates a positively transfected CA1 neuron. **(B)** Magnified view of transfected CA1 neuron. **(C,C’)** Representative images showing the expression of Tenm3 and GFP in a basal dendritic segment of the CA1 neuron from panel **(B)**. Dendritic spines are indicated by yellow ROIs. Note the different Tenm3 levels in neighbouring spines, with some spines expressing Tenm3 (filled arrows) and some devoid of Tenm3 expression (empty arrows). **(D)** Proportion of SAPs with high and low Tenm3 signal below basal and apical Tenm3-positive and -negative spines. **(E)** Correlation of SAP- and spine-Tenm3 intensity in basal (purple) and apical (red) dendrites. *r*, non-parametric Spearman correlation coefficient. SAP: Spine Attachment Point. Scale bar, 200 μm **(A)**, 20 μm **(B)**, and 10 μm **(C)**.

Tenm3 is expressed in CA1 neurons of the hippocampus in a strong proximal-to-distal gradient along the CA1 region of the hippocampus (Berns et al., 2018). To further explore the subcellular localisation pattern of Tenm3 in CA1 neurons we biolistically co-transfected organotypic mouse hippocampal slice cultures with expression vectors for myc-tagged Tenm3 and membrane-GFP, allowing the subcellular detection of Tenm3 as well as visualisation of the overall cell morphology (Figures 3A,B). Our general analysis showed that Tenm3 is localised to dendritic spines in CA1 neurons with a total proportion similar to our *in vitro* assessment before (19.87% for both apical and basal spines combined, Supplementary Figure 4). Furthermore, we found strong differences in Tenm3 signal in spines within a few micrometres of each other along the same dendrite; some showed high levels of Tenm3 while neighbouring spines had barely detectable levels (Figures 3C,C’). Interestingly, Tenm3 was detected not only in the spine head, but also inside the dendritic shaft below the spines, referred to as the spine base or spine attachment points (SAPs; Supplementary Figure 5). In basal and apical dendrites where we found Tenm3 signals in the spine head, we also detected a clear signal in the SAPs in 71.95 and 93.02% of the cases, respectively (Figure 3D). There is a positive correlation between the Tenm3 signal detected in the spine head and the one in the SAPs (Figure 3E, *r* = 0.237, *p* < 0.0001 and *r* = 0.192, *p* = 0.0075 for basal and apical spines, respectively). However, we noted that there is a considerable number of Tenm3-negative spine heads that nevertheless showed strong Tenm3 signal at the SAPs (basal: 46.41%; apical: 56.33%, Figure 3D), pointing towards a possible involvement for Tenm3 in processes occurring at the shaft, including dendritic spine organisation.

### The intracellular domain of Tenm3 facilitates protein localisation to synaptic puncta

**FIGURE 4:**
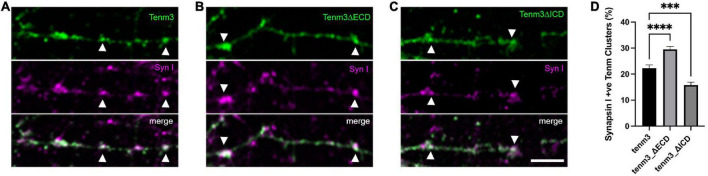
The intracellular domain of Tenm3 drives protein localisation to the synapse. Synapsin I immunostaining in dissociated cortical cells overexpressing **(A)** full-length, **(B)** ECD-deleted (Tenm3ΔECD), and **(C)** ICD-deleted (Tenm3ΔICD) variants of EGFP-tagged Tenm3 showing punctate expression for all cases. Arrow heads show two example for co-localisation in each panel. **(D)** Quantification of co-localisation with Tenm3ΔECD showing significantly higher (****p < 0.0001), and Tenm3ΔICD significantly lower (***p = 0.0004), co-localisation with synapsin I. Data represents mean ± SE based on samples with at least 37 cells/condition pooled from a least three different experiments. One-way ANOVA with Dunnett’s multiple comparison test was performed between all conditions (*p* < 0.05). Scale bar, 5 μm.

Based on our findings showing that Teneurins, when expressed in neurons, do not simply distribute evenly in the membrane, but appear in part in discrete puncta overlapping synaptic locations, we wondered how this specific localisation is achieved. Teneurins are large proteins with well-defined domains responsible for mediating a variety of interactions, but which domain, if any, is responsible for driving its synaptic localisation? To address this question, we created two different deletion mutants of our *tenm3* expression construct leading to different Tenm3 proteins: one lacking the extracellular domain (tenm3ΔECD) and one lacking the intracellular domain (tenm3ΔICD). In both constructs an EGFP-tag replaced the truncated domain to allow detection, while the original short transmembrane domain was kept present. We then assessed both mutants for (a) the general formation of discrete puncta and (b) the overlap of Tenm3 puncta with the synaptic marker synapsin I. After confirming that our deletion constructs are expressing efficiently and that the proteins are inserted into the membrane using N2a cells (Supplementary Figure 6) we transfected these different variants into dissociated rat cortical neurons. Surprisingly, we found that both variants, Tenm3ΔECD and Tenm3ΔICD, are still forming distinct puncta along neurites similar to full length Tenm3 (Figures 4A–C).

We then investigated further if these puncta co-localise to synaptic structures at comparable levels to the full-length protein or if the deletion of either the extra- or intracellular domain alter these values. Interestingly, we found that the intracellular domain alone (Tenm3_ΔECD) showed a 36% increase in co-localisation with synapsin I, while deletion of the intracellular domain resulted in a 32% reduction compared to the proportion for the full-length protein (Figure 4D). These results indicate that the Tenm3 intracellular domain plays an important role for synaptic localisation.

### All teneurin paralogues can form heterodimers with each other in *cis*

Teneurins act as *cis*-dimers and interact either homophilically or heterophilically in *trans* (Antinucci et al., 2013; Berns et al., 2018; Sando et al., 2019). It is generally established that these *cis*-dimers are generated through the assembly of two identical molecules of the same teneurin paralogue. However, there is only limited information on the ability of different full-length teneurin paralogues to form *cis*-heterodimers (Feng et al., 2002). To investigate this in more detail we used a co-immunoprecipitation approach.

**FIGURE 5:**
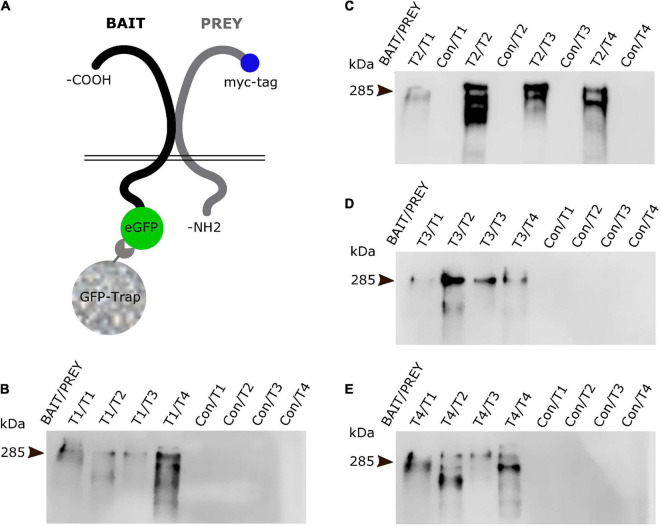
All teneurin paralogues can form heterodimers in *cis*. **(A)** Co-immunoprecipitation strategy with EGFP-tagged teneurin bait construct after co-transfection with tagRFP-tagged prey constructs in N2a cells. EGFP only expressing construct was used as control bait. Blots were probed for myc-tag which is only present on the prey constructs. **(B–E)** Bait teneurin paralogues were able to interact and specifically pull down itself and all other paralogues. Representative anti-myc-tag (prey) blots from **(B)** Tenm1, **(C)** Tenm2, **(D)** Tenm3 and, **(E)** Tenm4 pulldowns reveal presence of prey protein only in lanes with teneurin bait (Bait/Prey) but not with control bait (Con/Prey).

We generated EGFP-tagged versions of each teneurin paralogue as baits and tested if they were able to pull-down any of the other paralogues tagged with a myc-tag (prey, Figure 5A). We expressed both bait and prey proteins in N2a cells and used GFP-Trap beads for our pull downs and subsequent Western blot analysis. To detect the prey proteins, we used specific antibodies against the myc epitope. Both full-length protein bands and some proteolytic fragments can be detected. Interestingly, we found that all four teneurin paralogues (Tenm1 to Tenm4) used as bait were able to pull down all pray teneurin paralogues (Figures 5B–E). A Tenm3 variant with the EGF-like repeats removed did not result in the pull-down of any teneurin paralogue (data not shown). These results show, at least in an *in vitro* setting, that all teneurin paralogues are able to form heterodimers with each other and thus present different molecular *cis*-complexes for possible *trans*-interaction partners.

### Teneurin paralogues can be expressed in the same cell and are able to localise to different synaptic sites

**FIGURE 6:**
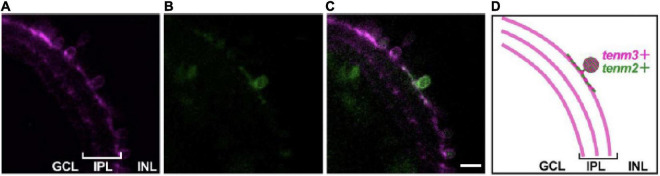
Teneurins are expressed together in the same cell *in vivo*. Tg (*tenm3:Gal4; UAS:RFP*) zebrafish embryos were injected with a *tenm2:citrine* BAC construct to mosaically label Tenm2-positive cells (citrine signal, **B**) in the background of all Tenm3-positive cells (RFP signal, **A**). **(C)** Merged image to show a small-field amacrine cell expressing both Tenm2 and Tenm3. **(D)** Schematic of the images showing the Tenm3- (tenm3+) and Tenm2 double-positive (tenm2+) amacrine cell. Scale bar, 10 μm.

**FIGURE 7:**
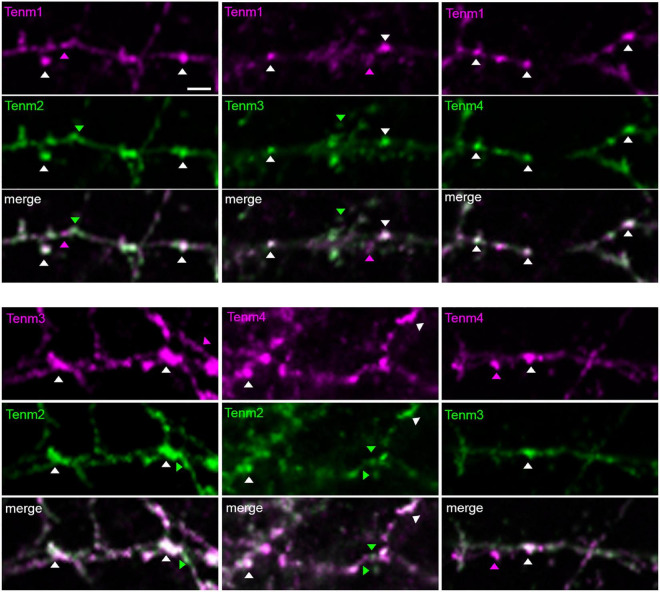
Different teneurin paralogues show overlapping localisation when expressed together in dissociated neurons. EGFP- and tagRFP-tagged teneurin paralogues co-transfected in primary neuronal cultures show partial co-localisation of puncta. Arrow heads show examples of puncta from individual paralogues and co-localisations. Scale bar, 20 μm.

To investigate the possibility of different teneurin paralogues being expressed in the same neuron *in vivo*, we used a combination of transgenic bacterial artificial chromosome (BAC) zebrafish lines to label teneurin-expressing cells (Cheung et al., 2019). Our results show indeed the presence of double labelled cells, for example, for Tenm2 and Tenm3 found in the retina (Figure 6). However, co-expression in the same neuron does not necessarily confirm co-localisation at synapses. We therefore co-expressed different fluorescently tagged teneurin paralogues in dissociated rat cortical neurons and assessed co-localisation using immunocytochemistry. Interestingly, we find that although most of the puncta along the neurites overlap between the different pairs of teneurin homologues (Supplementary Figure 7), we clearly find sites where only one or the other paralogue is localised (Figure 7). This suggests a possible mechanism to form different protein complexes and thus increases the molecular diversity at individual synapses.

## Discussion

Since their initial discovery in *Drosophila*, many studies have contributed to show the importance of teneurins in conferring neural connectivity and synaptic specificity across different species. While recent progress has been made in resolving the protein structure of teneuins, this has been focused on the large extracellular domain while the intracellular domain is still unresolved (Li et al., 2018; Jackson et al., 2019). Furthermore, other more basic biological characteristics of these proteins, for example, the *cis*-complex formation or localisation to different types of synapses, had so far not been systematically assessed. Here, we sought to answer some of these unanswered questions about teneurin biology by exploring the prevalence of teneurin at different synaptic sites, investigate factors driving its synaptic localisation, and further delve into the interchangeable interaction between different teneurin paralogues.

Using dissociated neurons and organotypic slice cultures, we found that while membrane-GFP on its own does not tend to cluster (Supplementary Figure 2C), all teneurins partially localise to individual puncta along axons and dendrites, overlapping well with synaptic markers. This is consistent with their important role during the formation of neural circuitry and specific synaptic interactions. The teneurin paralogues in *Drosophila*, Ten-m and Ten-a, are found localised to presynaptic, and mostly postsynaptic sites, respectively, and play a key role in neuromuscular synapse organisation (Mosca et al., 2012). In vertebrate cells, however, the pattern of teneurin presence across synapses was less clear. Previous data showed that Lasso, a splice variant of Tenm2, was localised mainly on dendrites in cultured neurons *in vitro* (Silva et al., 2011). Our findings on the synaptic localisation profile of all four teneurin paralogues in cultured neurons and comparison to other synaptic proteins strives to close this gap. Synaptic localisation was comparable between teneurin paralogues, with roughly a fifth (22%) of all teneurin protein puncta co-localising with the general presynaptic marker synapsin I. This apparently low proportion of co-localisation is however in line with our own and other previously published results for the synaptic proteins bassoon and shank2, which also show only a partial co-localisation with synapsin I (tom Dieck et al., 1998). Even the leucine-rich transmembrane protein LRRTM2, a key regulator and inducer of excitatory synapse development, is not fully co-localised with synapsin I when overexpressed in neurons, despite its potential to induce synapse formation (de Wit et al., 2009). Interestingly, we detected very good co-localisation of LRRTM2 and Tenm3. The fact that the majority (∼70%) of teneurin puncta did not co-localise with synapsin I is not completely unexpected, considering data for other synaptic cell adhesion molecules, such as cadherin 9 and αN-catenin, for example, which also form puncta along neurites and are enriched at synapses, but do not overlap perfectly with synaptic markers (Uchida et al., 1996; Williams et al., 2011). It is possible that this is either an effect from the overexpression strategy or that teneurins in general have additional roles along neurons, independent of the synaptic function. Further studies will be needed to ascertain the reasons behind this incomplete co-localisation.

The distribution of teneurin paralogues across different types of synapses showed that the localisation of teneurin to presynaptic puncta, whether this be excitatory or inhibitory, was similar (between 13 and 15% of puncta) and there was no significant difference between them. While the proportion of each teneurin paralogue at different sites is slightly lower than that of synapsin I, as expected, the combined total of teneurin puncta at excitatory and inhibitory presynaptic sites (28%) is, interestingly, a bit higher than the total observed colocalised with general presynaptic synapsin I. Similarly, while the overlap of teneurin is lowest with inhibitory postsynaptic sites (9%), paralogue localisation to excitatory postsynaptic sites is higher. The double-labelling of hippocampal sections for Tenm3 and synaptic markers in a recent study also shows that Tenm3 is more likely to be co-localised with excitatory synaptic markers (Zhang et al., 2022). On average, the total (excitatory and inhibitory) combined proportion of teneurin localised at either presynaptic (28%) or postsynaptic sites (29.5%) is almost equivalent, consistent for proteins that form homophilic complexes across the synaptic cleft.

Indeed, the slight variability in distribution, especially between teneurin either co-localised with synapsin I or combined vGAT and vGLUT, may be due to the natural heterogeneity of transfected neurons. Primary neuronal cultures consist of a heterogeneous population of cells with varying levels of pre-existing endogenous teneurin expression. This could therefore potentially lead to variations in overexpressed protein localisation. The direct epitope tagging of endogenous teneurins in neurons, *via* CRISPR gene editing for example, would circumvent this problem and is a possible alternative (Willems et al., 2020). Additionally, the co-localisation analysis method conducted indiscriminately accounts for all protein clusters, regardless of their localisation to axons or dendrites, and may represent protein clusters that are actively being transported along dendrites and axons. Although this transport is normally restricted to young neurons during the early stages of synapse formation, certain transport vesicles (or dense core vesicles), transporting the synaptic proteins piccolo and bassoon for instance, can be observed not only transporting to nascent presynapses in young neurons but also in mature synapses (Grabrucker et al., 2009). However, based on time-lapse imaging of transfected neurons, we could not detect any movement of the visible teneurin puncta (data not shown), therefore making this option unlikely.

The punctate and partial distribution of teneurin to synapses is reminiscent of Sema5B, which also only partially colocalises with PSD-95 and synapsin I (O’Connor et al., 2009). In addition to the cadherin-catenin complex, semaphorins have been shown to interact with the cytoskeleton and these interactions may be crucial for the regulation of synaptic connectivity and axon guidance, respectively (Salinas and Price, 2005; Pasterkamp and Giger, 2009; Wang et al., 2012). Teneurins have similarly been shown to be able to interact with the cytoskeleton, which further supports a role for teneurins in contributing to the establishment of synaptic connectivity, possibly through similar mechanisms (Nunes et al., 2005; Zheng et al., 2011; Mosca et al., 2012). It could also be that there are specific sites for local translation (for example, at the SAPs or elsewhere along the neurite) described for other synaptic proteins, which would generate a punctate pattern not necessarily overlapping with synaptic markers. However, currently it is not known if the mRNAs of teneurin paralogues are transported along neurites and would need further work.

We further explored the subcellular localisation of one of the teneurin paralogues, Tenm3. Using organotypic hippocampal slice cultures where the different areas can be directly recognised, we concentrated on neurons in the CA1 region, where Tenm3 is readily expressed. We found that tagged Tenm3 formed distinct puncta, overlapping with dendritic spines. Interestingly, Tenm3 was also found more frequently, and usually with higher relative signal intensity, to the SAPs below the spine neck, than inside the spine heads. This suggests that Tenm3 in the SAPs may potentially serve as a reserve pool for synaptic recruitment to the spine head, as has been observed for other proteins such as the cadherin-associated b-catenin and profilin, a regulator of actin polymerisation (Murase et al., 2002; Ackermann and Matus, 2003). The more abundant levels of Tenm3 at SAPs and their location at the base of spines also suggest a more general role for teneurins in synaptic assembly and organisation. The disruption of teneurins in invertebrates, for example, displays phenotypes consistent with a broad failure of synaptic organisation, such as failed active zone apposition, the disorganisation of synaptic proteins, and failure of pre- and postsynaptic differentiation (Mosca et al., 2012). The ability of teneurins to interact with the cytoskeleton (Nunes et al., 2005; Zheng et al., 2011; Mosca et al., 2012) suggests a role for Tenm3 protein in the SAPs in controlling synapse formation and assembly by arranging a cytoskeletal meshwork to regulate spatial organisation at the synapse.

Although we have demonstrated the localisation of teneurins to synapses, factors driving this localisation are still unknown. Our data shows that truncating full length Tenm3 significantly alters its ability to localise to synaptic sites. While a Tenm3 deletion mutant only containing the intracellular domain (Tenm3ΔECD) show an increased co-localisation with synapsin I, the mutant lacking this domain (Tenm3ΔICD) was significantly less likely to co-localise with synapsin I. These findings point towards a role of the intracellular domain in harbouring motifs that help bring teneurins to synaptic sites. While a synaptic targeting domain is known in some proteins, such as the short tyrosine-based motif followed by a pair of hydrophobic amino acids required for targeting PSD-95 protein to its postsynaptic localisation (Craven and Bredt, 2000), the potential synaptic localisation signal in the ICD of teneurin is currently unknown and will require further investigation. However, why is the deletion of such a domain not resulting in a complete abolishment of synaptic localisation? One possibility is that the extracellular domain alone is still able to form *cis*- and *trans*-dimers with endogenous, full-length proteins present at synapses and thus generate the GFP signal overlapping with synaptic staining. Future studies should show if the intracellular domains of the other teneurin paralogues also influence synaptic localisation.

Finally, we sought to further explore the possibility for heterodimeric interactions between different teneurin paralogues and what combinations were possible in *cis*. Recent studies utilising cell aggregation assays have shown that the ECD of Tenm4 is capable of interacting with the ECDs of all other teneurins (Hayashi et al., 2020). We show through co-immunoprecipitation experiments that *cis*-heterodimers can indeed form between all full-length teneurin paralogues *in vitro*. A control, where the EGF-like repeats crucial for *cis*-dimerisation were removed, failed to co-precipitate any protein partners in this assay. Although previous studies have demonstrated the possible heterodimerisation of the extracellular domain of different teneurin paralogues in HEK-293 cells (Feng et al., 2002), this is the first time the full *cis*-interaction matrix of all four full-length teneurin paralogues is presented. While further investigation is required over whether such interactions occur also *in vivo*, and under what circumstances, we can envisage a scenario where the precise combination of different teneurin paralogues is required for controlling synapse specificity in neuronal subpopulations. Heterodimer formation is common throughout biology as a simple way to increase functional diversity and the specific combination of proteins found within the synaptic area contributes to the molecular diversity of neuronal connections. However, for this to happen, different teneurin paralogues would have to be expressed in the same neuron. Our experiments in zebrafish showed that this is indeed the case. Combining this observation with our biochemical findings of detecting Tenm2/Tenm3 *cis*-interactions (as well as others), we speculate that such heterodimers indeed exist and play a role *in vivo*. However, our results based on the presence of different teneurins in dissociated neurons suggest that such *cis*-heterodimers are not necessarily a default mechanism in co-expressing cells. Interestingly, we find some puncta along neurites that seem to contain only one of the two paralogues present (Figure 7 and Supplementary Figure 7). Although this approach does not confirm the presence of teneurin *cis*-heterodimers, it does hint at a possible mechanism to change the molecular composition of individual synapses of a neuron and could be adding to the diversity of specific interactions in *trans*. Future more detailed molecular and biophysical assessment of such complexes and their underlying mechanisms should shine light on this central issue.

As important as the interactions in *cis*, the *trans*-heterophilic interactions of teneurin with the cell-surface protein latrophilin (LPHN) have been well documented in recent years and been shown to be critically involved in regulating synapse specificity (excitatory versus inhibitory), for example, through the alternative splicing of *tenm2* altering the Tenm2/LPHN adhesion complex (Li et al., 2020). It remains to be seen whether teneurin interactions in *trans* with other teneurins (either heterophilic or homophilic) are able to contribute to synaptic specificity in the same way in vertebrates as the orthologues in *Drosophila* (Hong et al., 2012; Mosca et al., 2012). Indeed, recent observations suggest that while teneurins are expressed both pre- and postsynaptically, they possibly function primarily as presynaptic adhesion molecules that interact with other postsynaptic ligands such as LPHNs and may not actually be required for postsynaptic interactions themselves (Zhang et al., 2022). This is because only the pre- but not postsynaptic deletions of Tenm3 and Tenm4 in the medial entorhinal cortex produced impairments in synaptic connectivity in mice (Zhang et al., 2022). Although this seems to contradict the *trans*-synaptic teneurin-teneurin interaction suggested by other studies, Zhang et al. observed this through studying only a subset of synapses, and teneurins may function distinctly in different neuron types, and as discussed earlier, are likely to be involved in other processes such as regulating aspects of synaptic organisation and homeostasis. Through our characterisation of the synaptic localisation of different teneurin paralogues, exploration of factors contributing to its localisation and identification of a novel way in which teneurins may increase molecular diversity through heterodimerisation, we make significant progress in expanding our understanding of this unique and multifaceted protein. In the future, it will be important to further discover in detail the necessary factors that influence the generation of different molecular complexes at the synapse, including teneurins, and thus control the generation of synaptic diversity in the nervous system.

## Data availability statement

The original contributions presented in this study are included in the article/Supplementary material, further inquiries can be directed to the corresponding author.

## Ethics statement

This animal study was reviewed and approved by the Animal Welfare and Ethical Review Body, King’s College London, and Home Office Project Licence PPL70/9036.

## Author contributions

RH, AC, and GS contributed to the conception and design of the study and finalised the manuscript. AC and GS performed the main localisation studies. AW performed the deletion construct localisation studies. AB carried out the statistical analysis. MM performed the zebrafish co-expression analysis. AC wrote the first draft of the manuscript. All authors contributed to the article and approved the submitted version.

## Supplementary material

Supplementary Figure 1 Tagged teneurin constructs are expressed at the cell membrane in HEK293 cells. EGFP-tagged teneurin constructs were transfected into HEK cells and imaged 48 h post transfection. All four EGFP-tagged teneurin paralogues could be found expressed at the membrane. Representative images showing Tenm1_EGFP **(A)**, Tenm2_EGFP **(B)**, Tenm3_EGFP **(C)**, and Tenm4_EGFP **(D)** expression. Scale bar, 20 μm.

Supplementary Figure 2 Co-localisation between LRRTM2, synapsin I and Tenm3. **(A)** Immunocytochemistry detection of synapsin I (green) in dissociated cortical cultures transfect with a construct to express LRRTM2 (magenta). **(B)** Neurons co-transfected with constructs to express LRRTM2 (magenta) and Tenm3 (green). **(C)** Control cultures transfected with a general construct to express EGFP showing no punctate pattern for EGFP (green) and no specific co-localisation with synapsin I (magenta). Scale bar, 5 μm.

Supplementary Figure 3 Representative image of the co-localisation analysis between bassoon and synapsin I in a dissociated hippocampal neuron. **(a)** Image of a representative hippocampal neuron stained for bassoon and synapsin I. Scale bar, 10 μm. **(b)** Dendritic segment of the neuron shown in panel **(a)**. Scale bar, 5 μm. **(c)** Dendritic segment stained for bassoon. **(d)** Dendritic segment stained for synapsin I. **(e)** Colocalisation between bassoon and synapsin I after image analysis. **(f)** ROIs around bassoon puncta after image analysis. **(g)** Binary image of synapsin I puncta after image analysis. **(h)** Control colocalisation between bassoon and synapsin I after 90° rotation of the bassoon image channel **(c,f)**. **(i)** ROIs around bassoon puncta after image channel was rotated 90°. **(j)** Binary image of synapsin I puncta after image analysis. **(k)** Co-localisation between bassoon and synapsin I (black) and synapsin I and bassoon-control (grey), ^****^*p* < 0.0001. **(l)** Proportion of bassoon clusters (black) and bassoon-control clusters (grey) co-localised with synapsin I per neuron, ^**^*p* = 0.0079.

Supplementary Figure 4 Histogram of basal (blue) and apical (red) spine Tenm3 intensity, where Tenm3 intensity is determined as the ratio Tenm3/GFP intensity. The dotted black line indicates the threshold, which spines are considered to express Tenm3 (1.531). Signal in spines below this threshold was considered to be background (calculated using the mean ration of intensity throughout the dendrite versus spine structures). This data indicates that that only a subpopulation of 17.34% of all basal spines and 25.5% of all apical spines are Tenm3-positive. When apical and basal spines are pooled together, the total proportion of Tenm3-positive CA1 spines amounted to 19.87%.

Supplementary Figure 5 Tenm3 localisation and analysis in dendritic SAPs of CA1 neurons. Representative image showing expression of Tenm3 **(A)** and GFP **(B)** in a basal dendritic segment. A merged image is shown in **(C)**, where for every image three ROIs were traced for analysis: spine ROI, SAP ROI, and background ROI. Scale bar, 1 μm.

Supplementary Figure 6 EGFP-tagged truncated teneurin variants are localised to the cell membrane in N2a cells. EGFP-tagged Tenm3 expression constructs were transfected into N2a cells and imaged 48 h post transfection. Both EGFP-tagged truncated Tenm3 variants could be found expressed at the membrane. Representative images showing Tenm3ΔECD_EGFP **(A)** and Tenm3ΔICD_EGFP **(B)** expression. Scale bar, 20 μm.

Supplementary Figure 7 Different teneurin paralogues show overlapping expression when expressed together in dissociated neurons. EGFP- and tagRFP-tagged teneurin paralogues co-transfected in primary neuronal cultures have similar patterns of expression and can be found in clusters along neurites. Scale bar, 20 μm.
