# Supplementary figures and images for: Teneurin paralogues are able to localise synaptic sites driven by the intracellular domain and have the potential to form cis-heterodimers
Source: Front Neurosci. 2022 Nov 3;16:915149. doi: 10.3389/fnins.2022.915149 (PMC9670113; doi:10.3389/fnins.2022.915149)

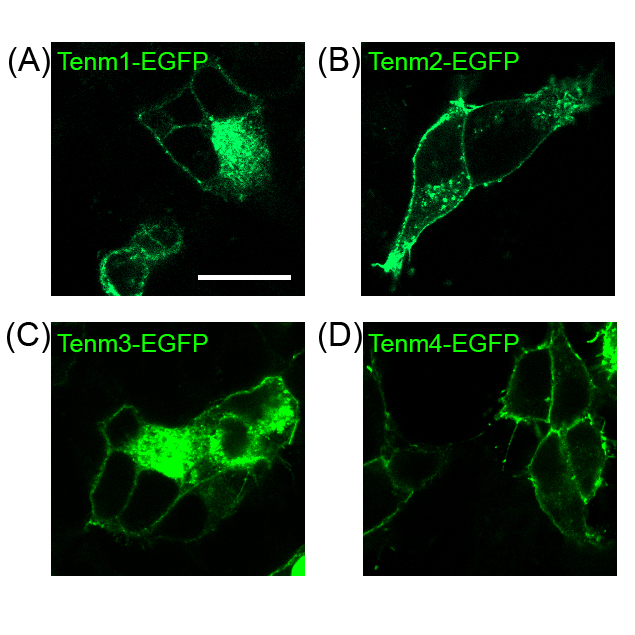

Supplement: Supplementary Figure 1 — Tagged teneurin constructs are expressed at the cell membrane in HEK293 cells. EGFP-tagged teneurin constructs were transfected into HEK cells and imaged 48 h post transfection. All four EGFP-tagged teneurin paralogues could be found expressed at the membrane. Representative images showing Tenm1_EGFP (A), Tenm2_EGFP (B), Tenm3_EGFP (C), and Tenm4_EGFP (D) expression. Scale bar, 20 μm. [file Image_1.JPEG]

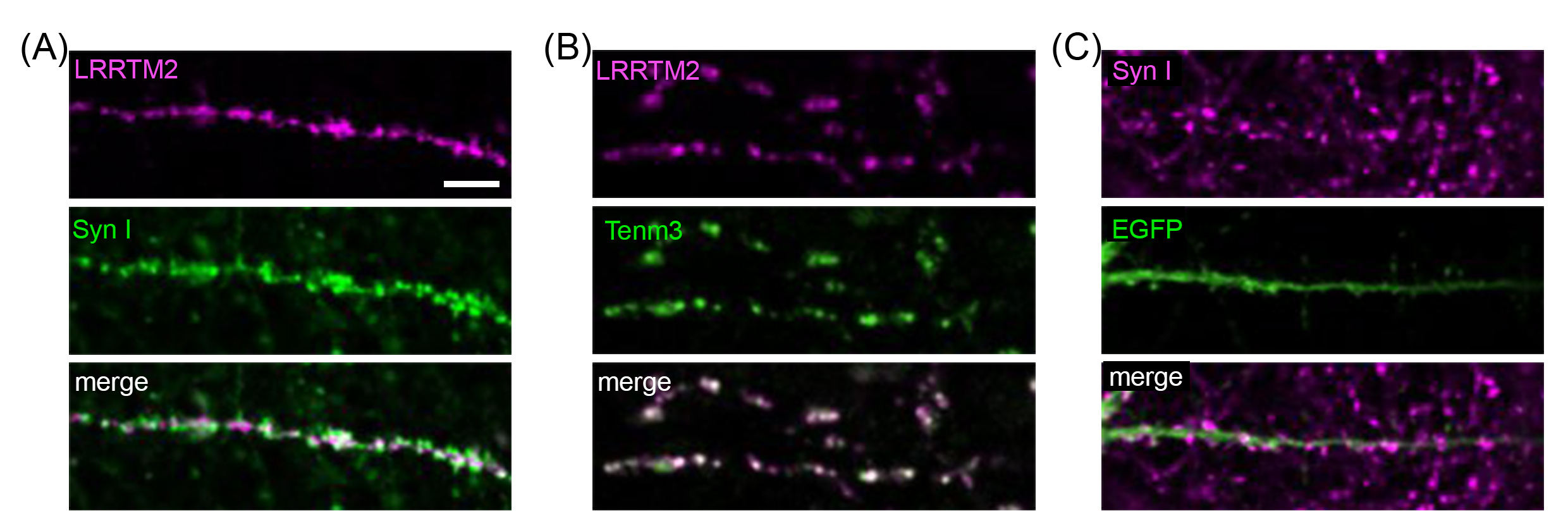

Supplement: Supplementary Figure 2 — Co-localisation between LRRTM2, synapsin I and Tenm3. (A) Immunocytochemistry detection of synapsin I (green) in dissociated cortical cultures transfect with a construct to express LRRTM2 (magenta). (B) Neurons co-transfected with constructs to express LRRTM2 (magenta) and Tenm3 (green). (C) Control cultures transfected with a general construct to express EGFP showing no punctate pattern for EGFP (green) and no specific co-localisation with synapsin I (magenta). Scale bar, 5 μm. [file Image_2.JPEG]

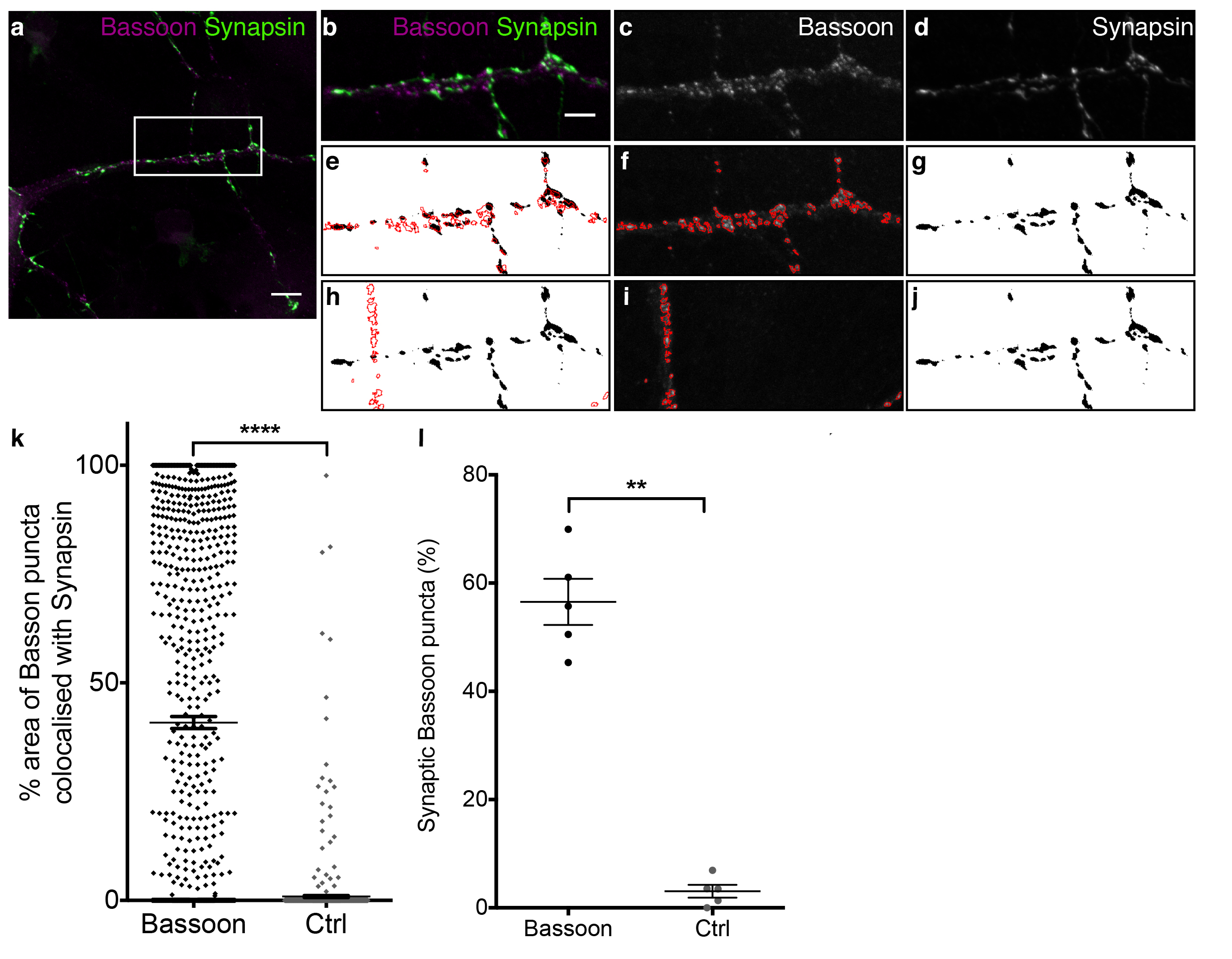

Supplement: Supplementary Figure 3 — Representative image of the co-localisation analysis between bassoon and synapsin I in a dissociated hippocampal neuron. (a) Image of a representative hippocampal neuron stained for bassoon and synapsin I. Scale bar, 10 μm. (b) Dendritic segment of the neuron shown in panel (a). Scale bar, 5 μm. (c) Dendritic segment stained for bassoon. (d) Dendritic segment stained for synapsin I. (e) Colocalisation between bassoon and synapsin I after image analysis. (f) ROIs around bassoon puncta after image analysis. (g) Binary image of synapsin I puncta after image analysis. (h) Control colocalisation between bassoon and synapsin I after 90° rotation of the bassoon image channel (c,f). (i) ROIs around bassoon puncta after image channel was rotated 90°. (j) Binary image of synapsin I puncta after image analysis. (k) Co-localisation between bassoon and synapsin I (black) and synapsin I and bassoon-control (grey), ****p < 0.0001. (l) Proportion of bassoon clusters (black) and bassoon-control clusters (grey) co-localised with synapsin I per neuron, **p = 0.0079. [file Image_3.JPEG]

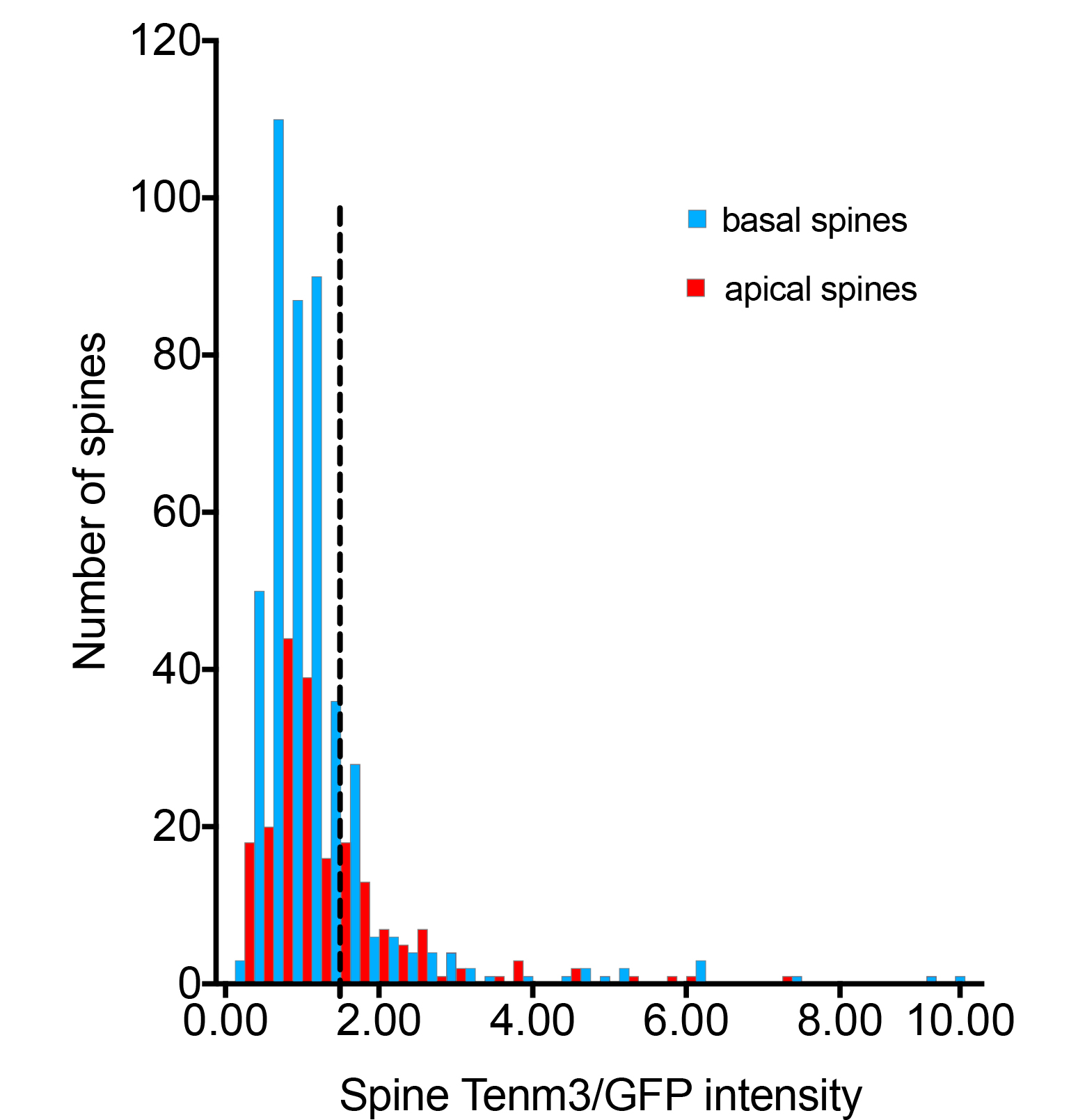

Supplement: Supplementary Figure 4 — Histogram of basal (blue) and apical (red) spine Tenm3 intensity, where Tenm3 intensity is determined as the ratio Tenm3/GFP intensity. The dotted black line indicates the threshold, which spines are considered to express Tenm3 (1.531). Signal in spines below this threshold was considered to be background (calculated using the mean ration of intensity throughout the dendrite versus spine structures). This data indicates that that only a subpopulation of 17.34% of all basal spines and 25.5% of all apical spines are Tenm3-positive. When apical and basal spines are pooled together, the total proportion of Tenm3-positive CA1 spines amounted to 19.87%. [file Image_4.JPEG]

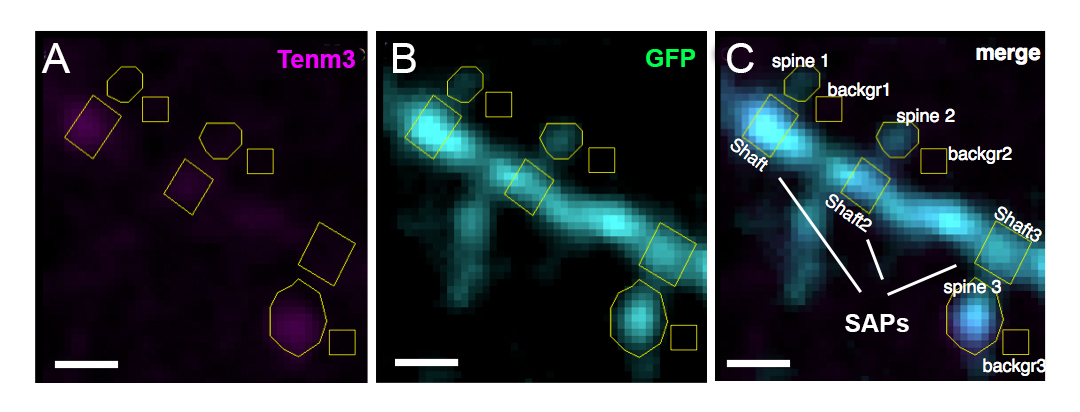

Supplement: Supplementary Figure 5 — Tenm3 localisation and analysis in dendritic SAPs of CA1 neurons. Representative image showing expression of Tenm3 (A) and GFP (B) in a basal dendritic segment. A merged image is shown in (C), where for every image three ROIs were traced for analysis: spine ROI, SAP ROI, and background ROI. Scale bar, 1 μm. [file Image_5.jpg]

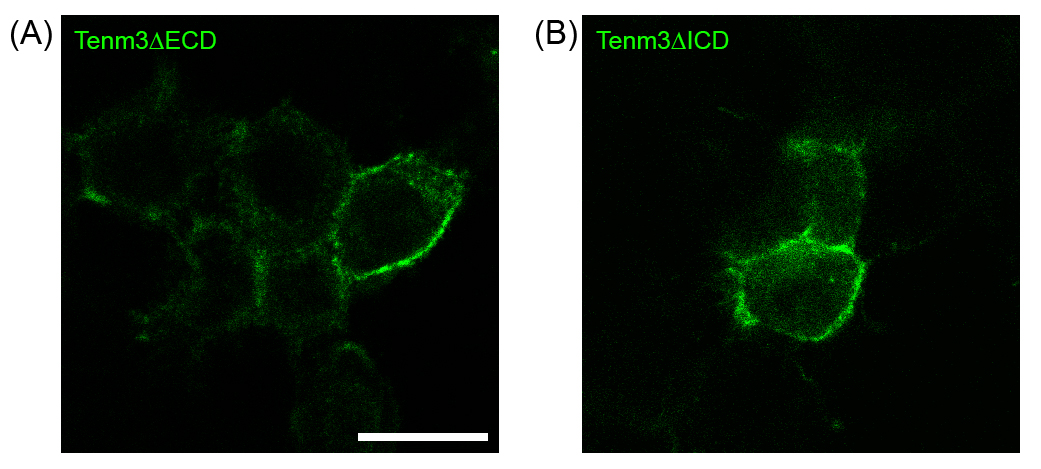

Supplement: Supplementary Figure 6 — EGFP-tagged truncated teneurin variants are localised to the cell membrane in N2a cells. EGFP-tagged Tenm3 expression constructs were transfected into N2a cells and imaged 48 h post transfection. Both EGFP-tagged truncated Tenm3 variants could be found expressed at the membrane. Representative images showing Tenm3ΔECD_EGFP (A) and Tenm3ΔICD_EGFP (B) expression. Scale bar, 20 μm. [file Image_6.JPEG]

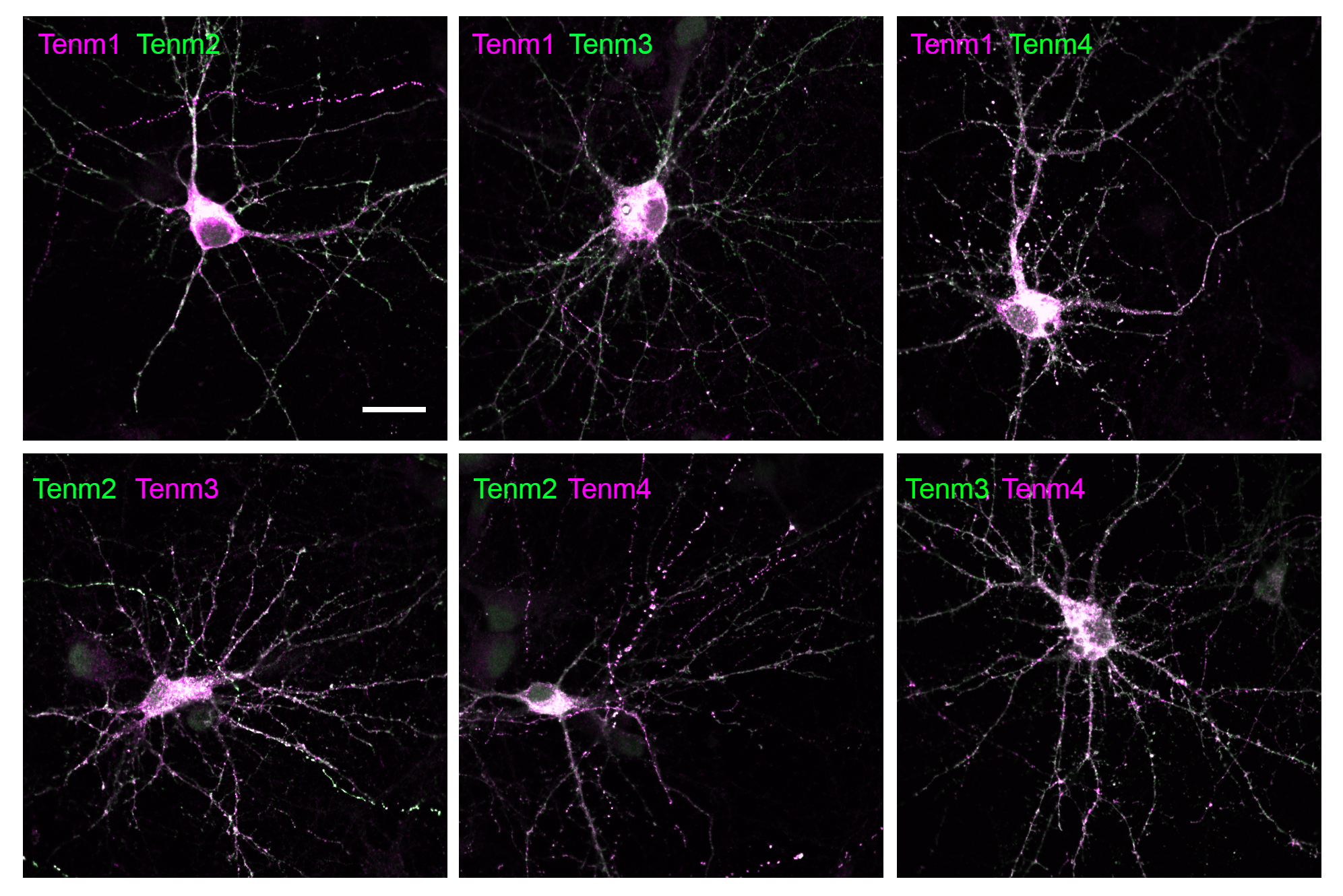

Supplement: Supplementary Figure 7 — Different teneurin paralogues show overlapping expression when expressed together in dissociated neurons. EGFP- and tagRFP-tagged teneurin paralogues co-transfected in primary neuronal cultures have similar patterns of expression and can be found in clusters along neurites. Scale bar, 20 μm. [file Image_7.JPEG]
